# Association between elevated serum matrix metalloproteinase-2 and tumor necrosis factor-α, and clinical symptoms in male patients with treatment-resistant and chronic medicated schizophrenia

**DOI:** 10.1186/s12888-024-05621-6

**Published:** 2024-03-01

**Authors:** Haidong Yang, Ruijie Peng, Man Yang, Jing Zhang, Zhihui Shi, Xiaobin Zhang

**Affiliations:** 1 Department of Psychiatry, The Fourth People’s Hospital of Lianyungang, The Affiliated KangDa College of Nanjing Medical University, 222003 Lianyungang, P.R. China; 2 Suzhou Psychiatric Hospital, Institute of Mental Health, The Affiliated Guangji Hospital of Soochow University, 215137 Suzhou, P.R. China

**Keywords:** Neuroinflammation, Matrix metalloproteinases-2, Tumor necrosis factor-α, Treatment-resistant schizophrenia

## Abstract

**Background:**

Inflammation has an important role in the pathogenesis of schizophrenia. The aim of this study was to investigate the levels of tumor necrosis factor (TNF) and matrix metalloproteinase-2 (MMP-2) in male patients with treatment-resistant schizophrenia (TRS) and chronic medicated schizophrenia (CMS), and the relationship with psychopathology.

**Methods:**

The study enrolled 31 TRS and 49 cm male patients, and 53 healthy controls. Serum MMP-2 and TNF-α levels were measured by the Luminex liquid suspension chip detection method. Positive and Negative Syndrome Scale (PANSS) scores were used to evaluate symptom severity and Repeatable Battery for the Assessment of Neuropsychological Status was used to assess cognitive function.

**Results:**

Serum TNF-α and MMP-2 levels differed significantly between TRS, CMS and healthy control patients (F = 4.289, *P* = 0.016; F = 4.682, *P* = 0.011, respectively). Bonferroni correction demonstrated that serum TNF-α levels were significantly elevated in CMS patients (*P* = 0.022) and MMP-2 levels were significantly higher in TRS patients (*P* = 0.014) compared to healthy controls. In TRS patients, TNF-α was negatively correlated with age (*r*=-0.435, *P* = 0.015) and age of onset (*r*=-0.409, *P* = 0.022). In CMS patients, MMP-2 and TNF-α were negatively correlated with PANSS negative and total scores, and TNF-α was negatively correlated with PANSS general psychopathology scores (all *P* < 0.05). MMP-2 levels were positively correlated with TNF-α levels (*P* < 0.05), but not with cognitive function (*P* > 0.05).

**Conclusion:**

The results indicate the involvement of inflammation in the etiology of TRS and CMS. Further studies are warranted.

## Introduction

Schizophrenia is a complex, heterogeneous, and chronically severe mental disorder characterized by a range of symptoms, including positive and negative symptoms, and cognitive deficits [1]. Despite advancements in pharmacological treatments, approximately one-third of patients will develop treatment-resistant schizophrenia (TRS), which fails to respond adequately to standard antipsychotic medications [2]. Additionally, a subset of patients with schizophrenia become chronic and require continuous medication, which can lead to protracted symptoms and a persistent burden of disease [3, 4]. However, the reasons behind the suboptimal treatment outcomes in these populations are not fully understood. Further explorations of the underlying pathophysiological mechanisms are needed.

Recent studies have suggested that inflammation may play a critical role in the etiology of schizophrenia [5, 6]. One of the key inflammatory cytokines implicated in this process is tumor necrosis factor-alpha (TNF-α); the level of TNF-α is altered in patients with schizophrenia [7]. TNF-α has an established role in the inflammatory response and has been associated with various psychiatric symptoms, such as affective symptoms and cognitive function, as well as being involved in the etiology of the acute and chronic phases of schizophrenia [8–10]. The relationship between TNF-α levels and the clinical manifestations of schizophrenia presents a potential avenue for understanding the inflammatory etiology of schizophrenia as well as variations in symptoms.

Matrix metalloproteinases (MMPs) constitute a group of zinc-dependent proteases involved in the processing of active factors, such as cell-surface receptors, neurotrophic factors, chemokines, and cytokines, as well as the regulation of neuroinflammation [11]. MMPs contribute to synaptogenesis, plasticity, and long-term potentiation, play a role in converting pro-TNF-α into mature secretory proteins, and disrupt the integrity of the blood-brain barrier (BBB) [12, 13]. MMP-2 is one of the cores of the 25 known MMP enzymes. The connection between MMP-2 and inflammatory cytokines suggests a complex interplay that may contribute to the neuropathological changes observed in schizophrenia [14]. For instance, interleukin-6 up-regulates the production of MMP-2, and MMP-2 activates TNF-α [15–17]. Nonetheless, there are limited studies of MMP-2 in patients with schizophrenia, particularly in the context of treatment-resistant and chronically medicated patients.

Cognitive deficits have been widely demonstrated in patients with schizophrenia at various stages, including cases without psychopharmacological treatment, first-episode psychosis, and TRS [18, 19]. Neuroimmunity has been implicated as an etiological factor in the cognitive deficits of patients with schizophrenia [20]. In patients with schizophrenia, TNF-α has been associated with various domains of cognitive function [7, 21]. Studies in mouse models have demonstrated that inhibition of MMP-2 expression by the increased integrity of the BBB diminishes the decline in cognitive function [22, 23]. Nevertheless, the exact etiology of cognitive deficits in patients with TRS remains unclear.

Therefore, exploring the interaction of TNF-α and MMP-2 in patients with TRS and chronic medicated schizophrenia (CMS) could provide insight into the mechanisms driving treatment resistance and chronicity. The hypothesis of this study is that TNF-α and MMP-2 levels are altered in patients with TRS and CMS, and correlate with psychopathological symptoms. The aims of this research are to investigate (1) whether the levels of TNF-α and MMP-2 in patients with TRS and CMS differed compared to healthy controls, and (2) the relationship between TNF-α and MMP-2 levels with clinical symptoms and cognitive function.

## Methods

### Subjects

This observational, cross-sectional study using a case-control design recruited a total of 31 TRS and 49 cm male patients from the Fourth People’s Hospital of Lianyungang. Five patients were treated with clozapine alone, and the rest were treated with typical or atypical antipsychotics, with the drug dose finally converted to a chlorpromazine-equivalent dose. The diagnosis of schizophrenia was determined using the Structured Clinical Interview DSM-IV. The inclusion criteria were male sex, age 18–60 years, Han Chinese ethnicity, and absence of anti-inflammatory or antibiotic medication for at least 4 weeks prior to enrolment.

A semi-structured questionnaire was used to collect general data from the patients, which included age, sex, education, smoking status, height, weight, duration of illness, and age of onset. During the same period, 53 healthy controls from the local community of Lianyungang were matched to the patient group in terms of age, sex, education, smoking, and body mass index (BMI), without a diagnosis in accordance with the Axis I criteria for a major disease. A family history of mental illness was excluded. Exclusion criteria for all participants were comorbidities of major medical morbidity, degenerative neurological disorders, endocrine system disorders, and alcohol or substance dependence. The health status of all participants was determined by physical examination and laboratory tests such as blood count, liver and renal function tests, glucose levels, and thyroid function.

Informed consent was given by all participants or their guardians. The protocol was approved by the Ethics Committee of the Fourth People’s Hospital of Lianyungang City.

### Clinical and cognitive assessment

The severity of clinical symptoms in patients with schizophrenia was assessed by two experienced psychiatrists using the Positive and Negative Syndrome Scale (PANSS). The correlation coefficient among the scores of PANSS exceeded 0.8. Cognitive function of the subjects was assessed using the Repeatable Battery for the Assessment of Neuropsychological Status (RBANS), which consists of five subtests assessing immediate memory, visuospatial/structural, verbal, attentional, and delayed memory [24]. The raw total scores of the subscales were subsequently processed into standardized scores. The RBANS has demonstrated good reliability and validity in patients with schizophrenia and in the Chinese population [25].

### Defining TRS and CMS

Patients with TRS were defined as those who had been poorly effective on two antipsychotics consecutively for more than 6 months, whose equivalent dose of chlorpromazine was > 600 mg/day, and who had scores ≥ 3 for each of the eight PANSS subscales (P1, P2, P3, N1, N4, N6, G5, and G9) [26–28]. CMS patients were those who had a stable treatment effect of one antipsychotic medication for more than 6 months, an equivalent dose of chlorpromazine of < 600 mg/day, score < 3 for each of the eight aforementioned PANSS subscales, and a total PANSS score < 60 [26, 28].

### Measurement of serum MMP-2 and TNF-α levels

Fasting peripheral blood samples were drawn from each participant between 7:00 and 9:00 a.m. Each sample was centrifuged at 3500 rpm for 15 min and stored at -80 °C until analyzed. Clinical symptoms and cognitive function were performed after obtaining blood samples. Serum MMP-2 and TNF-α levels were measured using the Luminex liquid suspension chip detection method according to product instructions (R&D Systems, Minneapolis, MN, USA). All blood samples measurements were made in duplicate by a technician blinded to the clinical implications of the samples. The intra- and inter-assay variability of MMP-2 and TNF-α measurements was 2.05% and 2.93%, respectively.

### Statistical analyses

SPSS version 19.0 (IBM, Armonk, NY, USA) was used for statistical analyses, The Kolmogorov-Smirnov test was performed to assess the normal distribution of the variables. Continuous variables were analyzed using analysis of variance (ANOVA). Results are expressed as mean ± standard deviation. Variables that were not normally distributed were analyzed using the Mann–Whitney U-test and expressed as median with 25th and 75th quartiles. Categorical variables were tested using chi-square test. Differences were considered statistically significant at *P* < 0.05. Analysis of covariance (ANCOVA) was performed with TNF-α and MMP-2 as the dependent variables, with diagnosis as fix factor, age, BMI, smoking, and education as covariates. Bonferroni correction was performed for multiple testing, the Bonferroni-corrected P-value between the TRS and CMS groups was the original P-value × 4 for each of the PANSS scores. Cohen’s d value determined effect size. Pearson’s or Spearman’s correlation analysis was performed to explore the relationship between variables. Stepwise multiple regression was performed to determine the relationship between variables after controlling for confounding factors of age, education, smoking, chlorpromazine equivalent dose, duration of illness, and age of onset.

## Results

### Sample characteristics

Comparison of demographic data, clinical symptoms, and cognitive function between TRS, CMS, and healthy control groups are shown in Table 1. The range of years of education in the patient and healthy groups was 6–16 years. No significant differences were evident in age, education, BMI, and smoking status among the three groups (all *P* > 0.05). Differences in age of onset were not significantly different between the TRS and CMS groups (*P* = 0.236). Significant differences included the duration of illness, PANSS total scores and scores of the subscales, and the RBANS total scores and differences of the subscales (all *P* < 0.05). The Bonferroni post-hoc test showed that both the RBANS total and index scores TRS and CMS groups in the TRS and CMS groups were significantly lower than those of the healthy control group (all *P* < 0.05), with no significant difference found between TRS and CMS groups (all *P* > 0.05). Post-hoc tests revealed statistically significant differences between the TRS and CMS groups in the overall and subscale PANSS scores (all *P* < 0.05).

**Table 1:** Sociodemographic and clinical data of TRS and CMS patients, and healthy controls

	TRS (*n* = 31)	CMS (*n* = 49)	Controls (*n* = 53)	F/Z/χ^2^	*P*
Age (years)∗	40.65 (9.18)	40.61 (10.27)	40.02 (9.28)	0.063 ^a^	0.939
Education (years)∗	8.58 (2.53)	9.43 (3.02)	9.83 (3.06)	1.783 ^a^	0.172
BMI (kg/m^2^)∗	24.79 (3.80)	24.33 (3.60)	25.92 (3.07)	2.856 ^a^	0.061
Smoking (n, %)	12 (38.7%)	29 (59.2%)	20 (37.7%)	5.551 ^b^	0.062
Age of onset (years)∗	25.58 (7.73)	27.88 (8.76)	-	1.428 ^a^	0.236
Duration of illness (years)◊	13 (10, 22)	10 (7, 15)	-	− 1.98 ^c^	0.048
PANSS total score∗	74.03 (7.35)	47.55 (7.96)	-	222.819^a^	< 0.001
*P* subscores∗	14.03 (5.60)	9.12 (2.22)	-	30.353 ^a^	< 0.001
N subscores∗	35.06 (4.64)	25.55 (4.19)	-	89.962 ^a^	< 0.001
G subscores∗	24.94 (3.75)	13.33 (4.76)	-	132.125^a^	< 0.001
Equivalent dose of chlorpromazine (mg/d)◊	675 (615, 825)	500 (430, 565)	-	− 7.51 ^c^	< 0.001
RBANS total score∗	55.87 (9.98)	59.98 (11.18)	91.02 (11.83)	136.918^a^	< 0.001
Immediate∗ memory∗	47.39 (11.99)	52.24 (19.10)	86.85 (17.06)	75.428 ^a^	< 0.001
Visuospatial/ constructional∗	66.32 (15.46)	72.06 (14.64)	91.47 (15.21)	34.272 ^a^	< 0.001
Language∗	69.45 (12.80)	73.61 (14.41)	99.15 (10.29)	76.009 ^a^	< 0.001
Attention∗	79.16 (12.45)	84.16 (14.13)	108.72 (14.02)	60.735 ^a^	< 0.001
Delay memory∗	52.90 (15.02)	55.02 (17.04)	87.26 (16.28)	65.847 ^a^	< 0.001
TNF-α (pg/ml)∗	5.84 (4.57)	6.07 (4.77)	3.86 (2.99)	4.289 ^a^	0.016
MMP-2 (ng/mL)∗	31.67 (3.05)	30.93 (2.92)	29.55 (3.63)	4.682 ^a^	0.011

BMI, body mass index; PANSS, Positive and Negative Syndrome Scale. RBANS, Repeatable Battery for the Assessment of Neuropsychological Status; TNF-α, tumor necrosis factor-alpha; MMP-2, Matrix metalloproteinases. ∗ mean (SD), ◊ median (interquartile range); a, one-way of analysis of variance; b, *χ*^2^ test; c, Mann–Whitney *U*-test

### Serum TNF-α and MMP-2 levels in TRS and CMS patients, and healthy controls

**Fig. 1:**
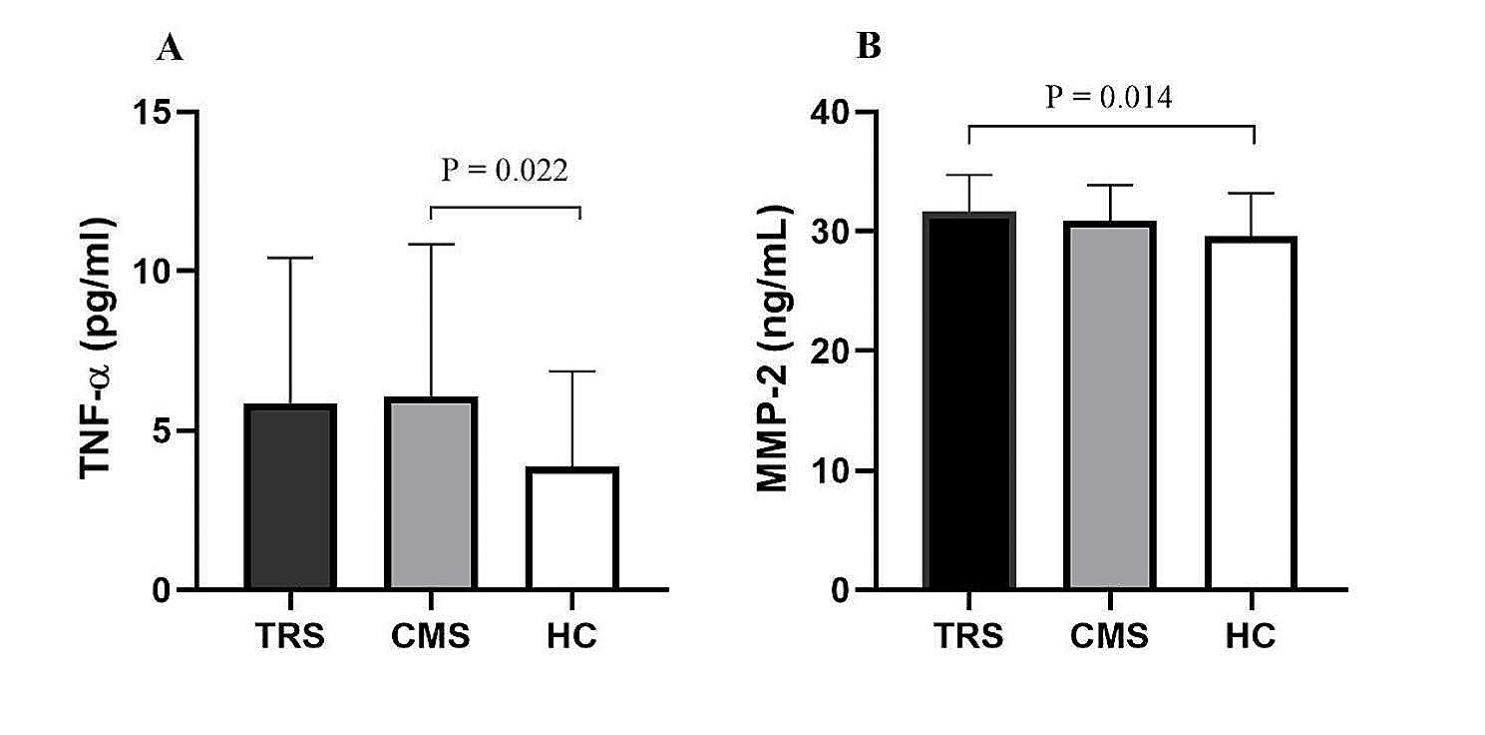
Comparison of serum TNF-α and MMP-2 levels in patients with TRS and CMS, and healthy controls

Serum TNF- α and MMP-2 levels varied significantly across patients with TRS and CMS, and the healthy controls (F = 4.289, *P* = 0.016; F = 4.682, *P* = 0.011, respectively). After controlling for age, BMI, smoking and education, ANCOVA revealed significant differences in the levels of TNF-α (F = 4.505, *P* = 0.013) and MMP-2 (F = 5.490, *P* = 0.005) between the TRS and CMS patients, and healthy controls. Bonferroni correction showed that TNF-α levels were increased with significant variances between CMS and healthy controls (*P* = 0.022). There was no significant difference between TRS and healthy controls (*P* = 0.104). The post-hoc results showed that elevated serum MMP-2 levels were significantly different between TRS and healthy controls (*P* = 0.014), but not between CMS and TRS patients (*P* = 0.986), and healthy controls (*P* = 0.10) (Fig. 1A and B).

### Association of MMP-2 and TNF-α levels with psychopathological and cognitive function in patients with TRS

**Fig. 2:**
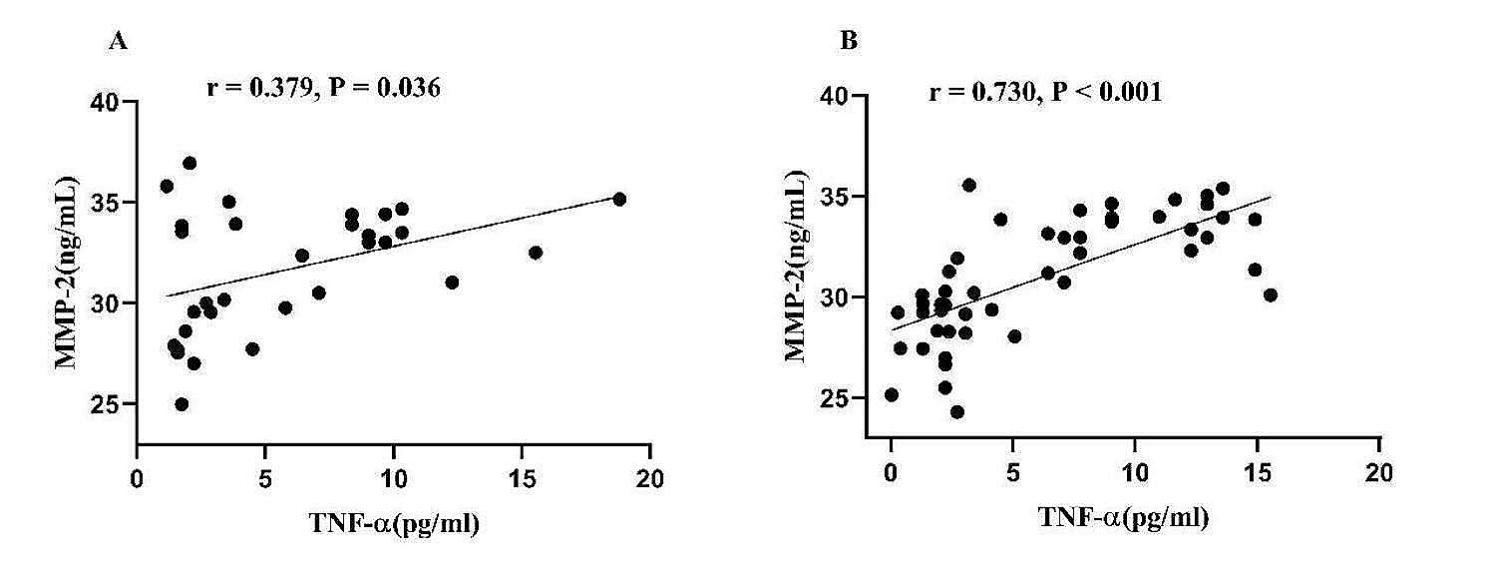
Relationship of serum of TNF-α and MMP-2 levels in patients with TRS (**A**) and CMS (**B**)

In patients with TRS, correlation analysis revealed that serum MMP-2 levels were positively associated with TNF-α (*r* = 0.379, *P* = 0.036) (Fig. 2A) and duration of illness (*r* = 0.438, *P* = 0.014). TNF-α was negatively related to age (*r* = − 0.435, *P* = 0.015) and age of onset (*r* = − 0.409, *P* = 0.022), after controlling for age and age of onset. There was no correction between MMP-2, TNF-α, and the PANSS total score and subscales, or the RBANS total score and subscales (all *P* > 0.05).

Subsequently, after controlling for confounders of age, education, smoking, and chlorpromazine equivalent dose, BMI, age of onset, and duration of illness, stepwise multiple regression demonstrated that MMP-2 was correlated with TNF-α (B = 0.628, t = 2.487, *P* = 0.019).

### Association of MMP-2 and TNF-α levels with psychopathological and cognitive function in patients with CMS

**Fig. 3:**
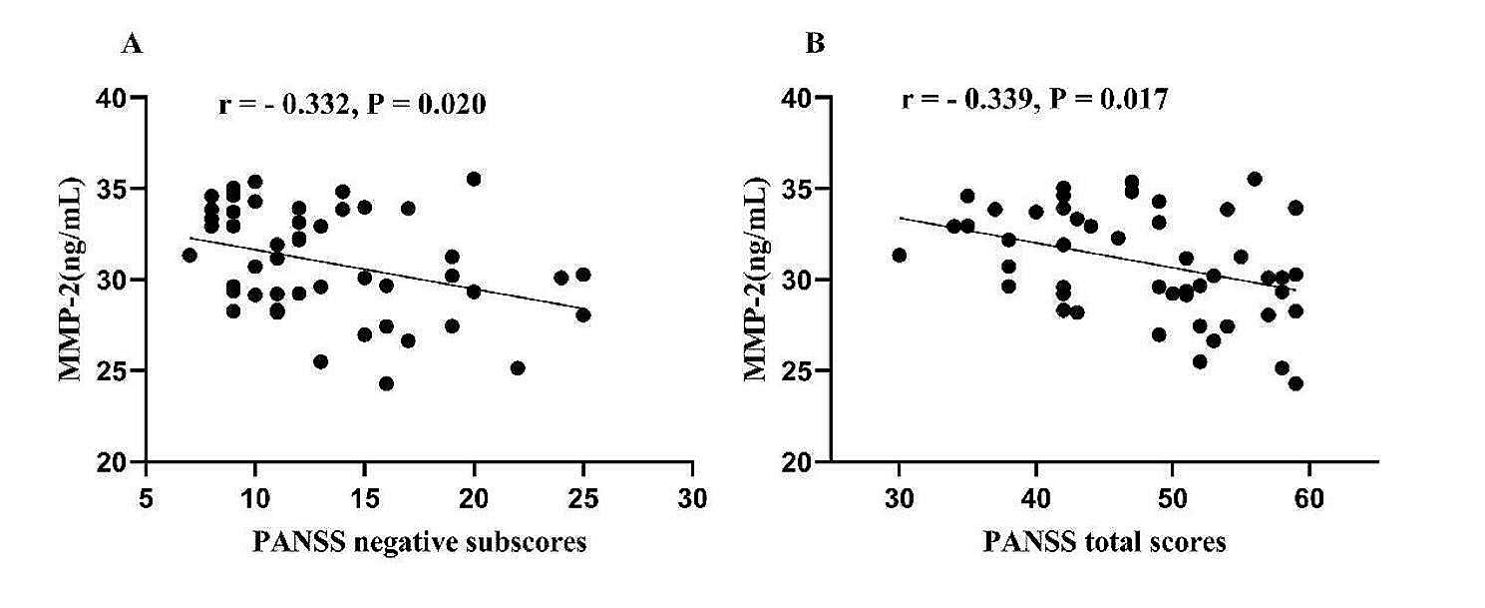
The relationship between MMP-2 and PANSS negative subscores (**A**) and total scores (**B**) in patients with CMS

In patients with CMS, correlation analyses showed that serum MMP-2 levels were positively correlated with TNF-α (*r* = 0.730, *P* < 0.001) (Fig. 2B) and negatively correlated with the PANSS negative subscores (*r* = − 0.332, *P* = 0.020) (Fig. 3A) and total scores (*r* = − 0.339, *P* = 0.017) (Fig. 3B). TNF-α levels were negatively correlated with the PANSS general psychopathology scores (*r* = − 0.288, *P* = 0.045), negative subscores (*r* = − 0.424, *P* = 0.002), and total scores (*r* = − 0.385, *P* = 0.006). No association was observed between MMP-2 and TNF-α with the RBANS total score and subscales (all *P* > 0.05).

After controlling for confounding factors with stepwise multiple regression analyses, MMP-2 levels were associated with TNF-α (B = 1.137, t = 6.640, *P* < 0.001), PANSS negative subscores (B = − 0.573, t = − 2.570, *P* = 0.013), and total scores (B = -1.018, t = -2.756, *P* = 0.008). TNF-α levels were associated with PANSS negative subscores (B = -0.344, t = − 2.515, *P* = 0.015), total scores (B = − 0,748, t = − 3.429, *P* = 0.001), and smoking (B = − 3.259, t = − 2.953, *P* = 0.005) as influencing factors between TNF-α (B = − 0.298, t = − 2.597, *P* = 0.013) and PANSS general psychopathology subscores.

## Discussion

There are four main findings in our study. First, serum MMP-2 levels were significantly elevated in patients with TRS compared to healthy controls, whereas serum TNF-α was significantly increased in patients with CMS. Second, MMP-2 and TNF-α levels were positively correlated in both patients with TRS and CMS. Third, in patients with CMS, TNF-α and MMP-2 levels were negatively correlated with the PANSS negative subscores and total scores, and TNF-α was negatively correlated with the PANSS general psychopathology subscores. Fourth, no correlation was evident between TNF-α and MMP-2 and cognitive function. To the best of our knowledge, this is the first study to reveal the relationship between elevated MMP-2 and TNF-α, clinical psychopathological symptoms, and cognitive function in TRS and CMS patients.

The finding that serum MMP-2 levels were elevated in patients with TRS is not entirely consistent with previous results. For example, Shibasaki et al. reported that MMP-2 levels were not different from those of healthy controls in a study involving 13 patients with schizophrenia and did not vary before or after electroconvulsive therapy [29]. Omori et al. demonstrated significantly elevated levels of MMP-2 in the cerebrospinal fluid of patients with schizophrenia [30]. The different findings may be related to sample size, ethnicity, source of biological samples, and diagnosis. Previous studies have shown that MMP-2 can increase the permeability of the BBB in mouse models [31, 32]. The significant elevation of serum MMP-2 levels in TRS patients could indicate a particular pathological process in this subgroup. MMP-2 play an important role in degrading the extracellular matrix and modulation of the BBB permeability may contribute to a neuroinflammatory state that underpins resistance to treatment.

In addition, our findings reveal the negative correlation of serum MMP-2 levels with PANSS negative scores and total scores in patients with CMS. A previous study found that cerebrospinal fluid MMP-2 levels were positively associated with core symptoms, psychogenic anxiety, and somatic anxiety in patients with major depressive disorder, but no correlation between cerebrospinal fluid MMP-2 and psychopathological symptoms was observed in patients with schizophrenia [30]. MMP-2 has been implicated in neuroinflammatory processes and neurodegeneration. In particular, MMP-2 modulated the integrity of the BBB and synaptic plasticity, which were posited to underlie certain aspects of schizophrenia pathophysiology [33, 34]. The negative symptoms, characterized by social withdrawal, apathy, and anhedonia, are often resistant to current pharmacotherapies and are linked to poorer functional outcomes [35, 36]. Our findings align with the hypothesis that aberrant neuroimmune activity, reflected in altered MMP-2 serum levels. The negative correlation could indicate that higher MMP-2 levels are associated with increased BBB permeability or impaired synaptic remodeling, potentially leading to exacerbated symptoms.

The major role of TNF-α in the pathophysiology of schizophrenia at various stages has been reported several times in previous studies, including in animal models [37–39]. Our study is consistent with previous findings that serum TNF-α levels were elevated in patients with CMS, suggesting a relationship between neuroinflammation and schizophrenia. Furthermore, the increase in TNF-α levels and their association with severity of symptoms as measured by PANSS suggest that ongoing inflammatory processes continue to play a role, even in the context of chronic medication. These findings are consistent with previous observations that persistent inflammation can contribute to the symptom burden in schizophrenia, despite ongoing treatment efforts [40, 41].

Previous studies have shown that patients with TRS not only manifest resistance to conventional dopamine D2 receptor blockers, but may also have abnormalities in glutamate regulation and altered cytokine levels [42, 43], which may combine to contribute to the complexity of the disease and the difficulty of treatment. In addition, prolonged use of dopamine D2 receptor blockers may lead to hypersensitivity of dopamine receptors, which may increase the relapse rate after discontinuation of the drug [44]. TNF-α also mediates glutamatergic activity by inducing α-amino-3-hydroxy-5-methyl-4-isoxazolepropionic acid receptor [45], and the TNF-NFκB-p53 axis restrict dopamine neuron survival in vivo [46], whereas the dopamine and glutamatergic systems have extensive and complex interactions [47]. Although our results indicate that the difference between TNF-α levels in TRS patients and healthy controls was not significant, based on the complex pathophysiology of TRS, we still could not exclude that TNF-α might be involved in the generation of TRS, nor could we rule out the influence of medications on this outcome, and further studies are needed.

Nonetheless, our findings revealed an unexpected negative correlation of MMP-2 and TNF-α with PANSS scores in CMS patients. This is inconsistent with the correlation of TNF-α with PANSS scores reported in previous studies. For example, TNF-α was found to be positively correlated with PANSS negative scores in 47 antipsychotic-responders and 47 antipsychotic non-responders with schizophrenia [48]. In contrast, in patients with schizophrenia who were treated for the first time [49], acutely exacerbated [50], displayed acutely relapsed states [51], and who were medicated in the acute phase [52], TNF-α did not correlate with PANSS scores. In paranoid patients with schizophrenia [40], TNF-α was negatively correlated with PANSS positive scores. In patients with chronic schizophrenia, TNF-α was negatively correlated with PANSS general pathology symptoms and total scores [41]. The relationship between MMP-2 and the severity of clinical symptoms in schizophrenia has not been reported. We speculate that long-term use of antipsychotics may have induced pharmacologic chronic immunomodulatory changes in the present study population. This may be reflected in altered levels of cytokines, which alter the routinely observed relationship between the cytokines and psychiatric measures. This finding emphasizes the complexity of the effects of long-term drug therapy on immune function and may provide new insights into biomarkers and assessment of treatment efficacy in patients with schizophrenia. Future studies should take these factors into account to provide a more nuanced exploration of the immune response in patients on long-term medication and its link to psychiatric severity. Interestingly, we found that serum TNF-α levels were associated with age of onset in patients with TRS. Xiu et al. reported that TNF-α gene − 1031T > C polymorphism was related to age of onset in patients with schizophrenia, suggesting that the TNF-α gene may serve as a modifier of age of onset in schizophrenia [53].

Previous studies have uncovered that MMP-2 and TNF-α have important roles in the progression of other diseases, such as Alzheimer’s disease and Parkinson’s disease [54, 55]. Mouse models have revealed that MMP-2 serves as a sheddase for TNF-α [56], suggesting that synergistic effects of MMP-2 and TNF-α may result in increased neuroinflammation and neurol destruction. The other major finding of the present study was the positive correlation between MMP-2 and TNF-α levels observed in patients with TRS and CMS. This finding may provide new insights into the shared inflammatory pathways that possibly contribute to modifying the pathological process. This interrelation warrants further investigation.

In previous studies, high expression of the MMP-2 gene on mRNA and protein levels in recurrent depressive disorders reported positive effects on cognitive efficiency, such as working memory and executive and attentional functions [57], and inhibition of MMP-2 attenuated cognitive impairment in aged mice [22]. A recent systematic review and meta-analysis indicated the correlation between TNF-α and cognitive deficits in schizophrenia [7]. However, the lack of correlation between MMP-2 and TNF-α and cognitive function in our study was intriguing and suggests that the cognitive deficits in TRS and CMS may not be mediated by a single mechanism. Rather, these deficits may also be related to the interaction or moderating effect between MMP-2 and TNF-α levels, and may also be related to the education of the patients, culture, and assessment instruments. Based on the positive effects of MMP-2 on cognitive efficacy in recurrent depression, how elevated MMP-2 levels in patients with schizophrenia influence cognitive deficits needs further investigation.

Limitations of this study should be acknowledged. Firstly, the cross-sectional design precludes causal inferences. Longitudinal studies are necessary to determine the temporal relationship between MMP-2 and TNF-α levels and clinical symptoms. Secondly, our sample size was relatively small, which may limit the generalizability of our findings. Thirdly, while we controlled for medication use, the heterogeneity in treatment regimens could have influenced the biomarker levels. Fourthly, selectivity errors may result based on defined TRS or CMS inclusion criteria and will need to be avoided in the future by increasing the sample size, introducing samples from multiple sources, and performing sensitivity analyses. Fifthly, participants were not assessed for psychotherapeutic or counseling interventions, which is one of the possible protective factors for non-pharmacological means of treatment that may have an important impact on the course of the illness and response to treatment for patients with schizophrenia [58, 59]. In addition, participants were not assessed for neuroimaging. Neuroimaging is becoming increasingly important in the complex diagnosis of psychiatric disorders and treatment follow-up [60–62]. The lack of neuroimaging limits our ability to gain a deeper understanding of the pathomechanisms of schizophrenia from a neurobiological perspective. Therefore, future studies should consider including these important aspects in order to understand more fully the treatment process of psychiatric disorders and to assess the treatment needs and responses of people with schizophrenia. Finally, the lack of cognitive correlation may be due to the cognitive measures used; future studies should include a broader assessment of cognitive function.

In conclusion, our study provides evidence for the involvement of MMP-2 and TNF-α in the pathology of TRS and CMS, with potential implications for the development of new treatment strategies. Future research should aim to elucidate the mechanisms by which these biomarkers influence the disease process and to explore their potential as targets for novel therapeutic interventions.

## Data Availability

The datasets used and analyzed during the current study are available from the corresponding author on reasonable request.
